# Effect of a systematic lung-protective protocol for COVID-19 pneumonia requiring invasive ventilation: A single center retrospective study

**DOI:** 10.1371/journal.pone.0267339

**Published:** 2023-01-12

**Authors:** Yoshihiko Takahashi, Shu Utsumi, Kenji Fujizuka, Hiroyuki Suzuki, Noritaka Ushio, Yu Amemiya, Mitsunobu Nakamura

**Affiliations:** 1 Advanced Medical Emergency Department and Critical Care Centre, Maebashi Red Cross Hospital, Gunma, Japan; 2 Department of Emergency and Critical Care Medicine, Graduate School of Biomedical and Health Sciences, Hiroshima University, Hiroshima, Japan; 3 Department of Emergency and Critical Care Medicine, Osaka Medical and Pharmaceutical University, Osaka, Japan; Bari University Aldo Moro, ITALY

## Abstract

The benefits of introducing a systematic lung-protective protocol for coronavirus disease 2019 (COVID-19) pneumonia requiring invasive ventilation in the intensive care unit (ICU) are unknown. Herein, we aimed to evaluate the clinical effects of introducing such a protocol in terms of mortality, duration of ventilation, and length of ICU stay. In this single-centre, retrospective, quality comparison study, we identified patients with COVID-19 pneumonia who received invasive ventilation in our ICU between February 2020 and October 2021. We established a systematic lung-protective protocol for the pre-introduction group until March 2021 and the post-introduction group after April 2021. Patients who did not receive invasive ventilation and who underwent veno-venous extracorporeal membrane oxygenation in a referring hospital were excluded. We collected patient characteristics at the time of ICU admission, including age, sex, body mass index (BMI), comorbidities, sequential organ failure assessment (SOFA) score, acute physiology and chronic health evaluation II (APACHE II) score, and Murray score. The study outcomes were ICU mortality, length of ICU stay, and duration of ventilation. The pre-introduction and post-introduction groups included 18 and 50 patients, respectively. No significant differences were observed in sex, BMI, SOFA score, APACHE II score, and Murray score; however, age was lower in the post-introduction group (70 vs. 56, P = 0.003). The introduction of this protocol did not improve ICU mortality. However, it reduced the ICU length of stay (26 days vs. 11 days, P = 0.003) and tended to shorten the duration of ventilation (15 days vs. 10 days, P = 0.06). The introduction of the protocol was associated with a decrease in the length of ICU stay and duration of ventilation; however, it did not change mortality. The application of the protocol could improve the security of medical resources during the COVID-19 pandemic. Further prospective multicentre studies are needed.

## Introduction

Coronavirus disease 2019 (COVID-19) is a pandemic that has spread at an overwhelming rate and has caused many deaths [1]. The severity of COVID-19 pneumonia varies, and most cases are mild and non-fatal [2, 3]. However, the most severe cases requiring invasive ventilation, which we called severe COVID-19 pneumonia, meet the criteria for acute respiratory distress syndrome (ARDS) and have a high fatality rate [4]. Since these patients require intensive care, each country needs to secure intensive care unit (ICU) resources and investigate effective treatments [5].

Severe COVID-19 pneumonia has been found to share many similarities with ARDS in terms of respiratory mechanics [6]. Therefore, lung-protective strategies, such as lung-protective ventilation with continuous intravenous infusion of a neuromuscular blocking agent, early application of prone positioning, and prolonged prone positioning, could be effective not only for patients with ARDS but also for those with severe COVID-19 pneumonia [7–10]. However, these measures have not been fully implemented. There is a need for the implementation of such measures in clinical practice [11].

We anticipated that it would be valuable to establish a systematic protocol for severe COVID-19 pneumonia and therefore implement a lung-protective strategy. The benefits of introducing a protocol for severe COVID-19 pneumonia remain unknown.

The aim of our study was to evaluate the clinical effects of a systematic lung-protective protocol for COVID-19 pneumonia in terms of mortality, duration of ventilation, and length of ICU stay.

## Methods

### Study setting

This was a single-centre, retrospective, quality comparison study conducted at Maebashi Red Cross Hospital. The study period was from February 2020 to October 2021, with the pre-introduction group until March 2021, and the post-introduction group after April 2021. Our study was approved by the Ethics Committee of the Maebashi Red Cross Hospital (ID: 2021–51), which waived the requirement for informed consent from patients and their relatives, given the retrospective and observational nature of the study. Our study is reported in accordance with the STrengthening the Reporting of OBservational studies in Epidemiology (STROBE) Statement (S1 Table).

### Population/patients

Patients over 18 years of age with COVID-19 pneumonia admitted to our ICU were screened. The exclusion criteria were the following: 1) patients who did not receive invasive ventilation, 2) patients who had contraindications for prone positioning (S2 Table), 3) and those who underwent veno-venous extracorporeal membrane oxygenation (V-V ECMO) in a referring hospital.

### Intervention/indicators

The systematic lung-protective protocol was introduced in patients requiring ventilator management with COVID-19 pneumonia admitted to our ICU from April 2021 to October 2021.

Firstly, after ICU admission, all patients were introduced to a high-flow nasal cannula, and oxygenation, respiratory acidosis, and respiratory effort were assessed.

The criteria for intubation were as follows: Patients met one of the conditions PaO_2_/F_I_O_2_ <150 mmHg [12], respiratory acidosis with pH<7.30, or excessive respiratory efforts.

Secondly, patients were placed in prone positioning with lung-protective ventilation and administered continuous intravenous infusion of rocuronium, a neuromuscular blocking agent, promptly after intubation. The target parameters of lung-protective ventilation were as follows: (i) tidal ventilation volume at 6 mL/ predicted body weight [13], (ii) driving pressure <15 cm H_2_O [14], and (iii) plateau airway pressure <30 cm H_2_O [15]. In addition, the positive end-expiratory pressure (PEEP) was determined according to the lower PEEP/higher F_I_O_2_ table proposed by the ARDS Clinical Trials Network [16]. The targets of prone positioning were as follows: (i) early prone positioning: the application of prone positioning within 36 h after intubation, and (ii) prolonged prone positioning: average duration being at least 16 h per session [7].

Thirdly, prone positioning was discontinued when PaO_2_/ F_I_O_2_ >200 mmHg could be maintained for more than 4 h in the supine position. It was discontinued using a neuromuscular blocking agent simultaneously when prone positioning was completed.

Even if PaO_2_/ F_I_O_2_>200 mmHg could not be achieved, the continuation of prone positioning was limited to 96 h after intubation, at which point the need for V-V ECMO was discussed [17].

Fourthly, patients were slowly weaned off the respiratory setting by adjusting the respiratory setting and sedatives and if necessary, using a neuromuscular blocking agent for respiratory effort and ventilation asynchrony.

**Fig 1:**
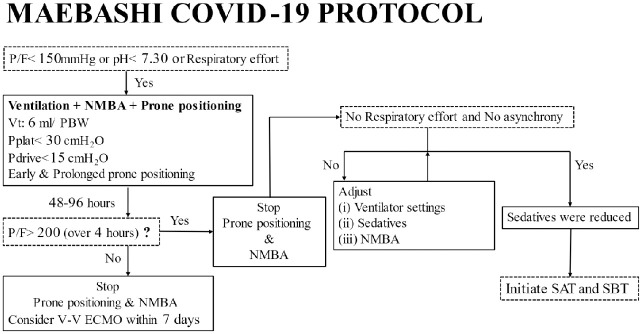
Maebashi COVID-19 protocol. MAEBASHI COVID-19 PROTOCOL (included the lung-protective strategy): Lung-protective ventilation with neuromuscular blocking agents, early application of prone positioning, and prolonged prone positioning. Abbreviations: P/F = PaO2/FIO2; PBW = predicted body weight; Vt = Tidal Volume (mL); Pplat = plateau airway pressure; NMBA = neuromuscular blocking agent; V-V ECMO = veno-venous extracorporeal membrane oxygenation; SAT = spontaneous awakening test; SBT = spontaneous breathing test.

Finally, the protocol was withdrawn if they met the criteria for the initiation of spontaneous awakening test and spontaneous breathing test [18]. In addition, the protocol was terminated when V-V ECMO was introduced (Fig 1).

Drug therapy during the protocol is shown separately in S3 Table.

### Comparison/control

The control group comprised patients requiring ventilator management with COVID-19 pneumonia admitted to our ICU from February 2020 to March 2021 prior to the introduction of the protocol.

### Outcome measures

The primary outcome was mortality in the ICU. The secondary outcomes were duration of ventilation and length of stay in the ICU.

Ventilation duration was defined as the absence of reintubation or the use of non-invasive ventilation within 48 h of extubation. For patients with tracheostomy, we defined weaning from the ventilator as the ability to breathe without assistance from a tracheostomy cannula for more than 24 h [7].

### Data collection

We collected data as follows: (i) patient characteristics at the time of ICU admission included the following: age, sex, body mass index, comorbidities, sequential organ failure assessment (SOFA) score, acute physiology and chronic health evaluation II (APACHE II) score, Murray score [19]; (ii) medications received during ICU stay; (iii) availability of treatment with V-V ECMO; (iv) ventilator parameters within two days after ICU admission (tidal ventilation volume/predicted body weight, driving pressure, plateau airway pressure, positive end-expiratory pressure), induction time and average duration time of prone positioning, and presence of complications associated with prone positioning; (v) the incidence of ventilator-associated pneumonia (VAP) as defined in previous literature [20].

These data were collected retrospectively from electronic medical records.

### Statistical analysis

Distributed continuous variables without a normal distribution were presented as median and interquartile range. Categorical data were summarised as numbers or percentages. For univariate analysis, the Mann–Whitney U test for continuous variables and Fisher’s exact test for categorical variables were used for comparison. For multivariate analysis, if the residues did not follow a normal distribution, they were log-transformed and subjected to multiple regression analysis, adjusting for significant covariates at baseline. Data were assumed to be missing at random, with no imputation or interpolation of the missing values.

Statistical tests were two-tailed, and statistical significance was set at p<0.05. All statistical analyses were performed using EZR [21], a graphical user interface for R (R Foundation for Statistical Computing, Vienna, Austria).

## Results

### Baseline characteristics

**Fig 2:**
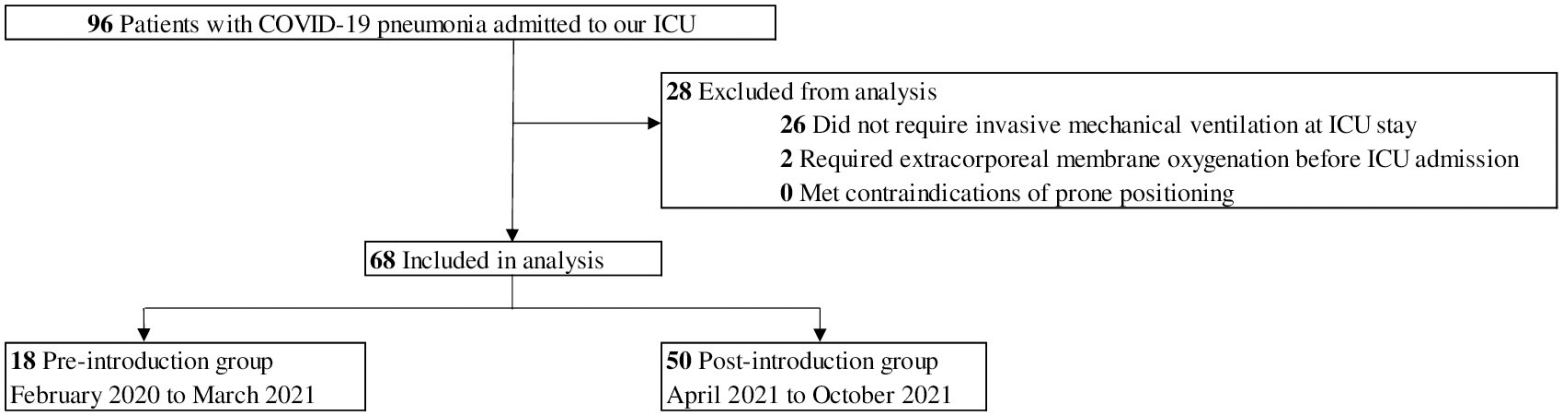
Study flow. We identified 96 patients over 18-years-old with coronavirus disease 2019 (COVID-19) pneumonia in our intensive care unit (ICU); 26 patients did not receive invasive ventilation, and 2 patients who underwent veno-venous extracorporeal membrane oxygenation in a referring hospital were excluded. A total of 68 patients were included in the analysis, with 18 in the pre-introduction group and 50 in the post-introduction group.

A total of 96 patients were admitted to our ICU, and of these, 68 patients who received invasive ventilation were eligible for our study (Fig 2). Eighteen patients were included in the pre-introduction group, and 50 patients were included in the post-introduction group.

Table 1 shows the patients characteristics.

**Table 1:** Baseline characteristics.

Characteristics	Overall (n = 68)	Pre-introduction (n = 18)	Post-introduction (n = 50)	P Value
Age (yr), median {IQR}	59 {52–70}	70 {61–74}	56 {49–65}	0.003
Gender (male), no./total no. (%)	42/68 (62)	10/18 (55)	32/50 (64)	
Body mass index (kg/m^2^), median {IQR}	26 {23–29}	25 {24–28}	26 {23–29}	
Acute Physiology and Chronic Health Evaluation II score, median {IQR}	26 {22–31}	29 {22–34}	25 {22–29}	
Sequential Organ Failure Assessment at ICU admission, median {IQR}	7 {5–9}	7 {3–9}	6.5 {6–9}	
Murray score, median {IQR}	2.5 {2.2–2.8}	2.5 {2.3–2.8}	2.5 {2.1–2.8}	
**Comorbidities, n (%)**				
Hypertension	22 (32)	11 (61)	11 (22)	0.002
Diabetes	19 (27)	4 (22)	15 (30)	
Renal failure	10 (15)	4 (22)	6 (12)	
Hepatic disease	3 (4)	1 (6)	2 (4)	
Coronary artery disease	5 (7)	1 (6)	4 (8)	
Cancer	6 (9)	1 (6)	5 (10)	
COPD	6 (9)	1 (6)	5 (10)	
Obesity (BMI>30)	14 (21)	3 (17)	11 (22)	
**Antiviral treatment, n (%)**				
Remdesivir	57 (84)	8 (44)	49 (98)	<0.001
Favipiravir	5 (7)	4 (22)	1 (2)	0.005
Lopinavir–Ritonavir	1 (1)	1 (6)	0	
Tocilizumab	3 (4)	0	3 (6)	
Baricitinib	16 (24)	0	16 (32)	0.006
Corticosteroids, no./total no. (%)	65/68 (96)	16/18 (89)	49/50 (98)	
Antibiotic treatment, no./total no. (%)	64/68 (94)	14/18 (78)	50/50 (100)	<0.001
VAP, no./total no. (%)	38/68 (56)	11/18 (61)	28/50 (50)	
Neuromuscular blocking agent, no. (%)	68 (100)	18 (100)	50 (100)	
V-V ECMO, no./total no. (%)	7/68 (10)	6/18 (33)	1/50 (2)	<0.001

Abbreviations: IQR = interquartile range; VAP = ventilator-associated pneumonia; V-V ECMO = veno-venous extracorporeal membrane oxygenation

There were no significant differences between the groups in sex, body mass index, SOFA score, APACHE II score, or Murray score; however, age was lower in the post-introduction group (70 vs. 56, *P* = 0.003). The type of antiviral drug used changed over time in the two groups, while the use of corticosteroids was not different. The incidence of VAP was not different between the two groups (OR, 0.64; 95% Cl, 0.18–2.16), although the use of antibiotics was higher in the post-introduction group (*P*<0.001). The rate of V-V ECMO significantly reduced from 33% to 2% in the post-introduction group (OR, 0.04; 95% Cl, 0.01–0.41).

### Lung-protective strategy

Table 2 shows the parameters of ventilation, prone positioning, and complications associated with prone positioning.

**Table 2:** Parameters of ventilation and prone positioning and complications related to prone positioning.

Ventilator parameters	Pre-introduction (n = 18)	Post-introduction (n = 50)	P Value
Vt/ PBW (ml/kg), median {IQR}	6.5 {5.8–6.8}	6.1 {5.6–6.4}	0.096
Pplat (cmH_2_0), median {IQR}	22 {20–24}	22 {22–23}	0.643
Pdrive (cmH_2_0), median {IQR}	12.5 {10–15}	12 {10–15}	0.92
PEEP (cmH_2_0), mean (SD)	10.3 (2.5)	10.7 (2.5)	0.590
**Prone positioning**			
Number of early prone positions performed, no./total no. (%)	10/18 (56)	49/50 (98)	<0.001
Time of prone per session, median {IQR}	15 {11–17}	18 {16–18}	0.007
**Complications related to prone positioning**			
Pressure ulcers, n (%)	0	2 (4)	
Endotracheal tube obstruction, n (%)	0	2 (4)	
Hemoptysis, n (%)	0	0	
Cardiac arrest, n (%)	0	0	
Oxygen saturation by pulse oximetry < 85% or PaO2 <55 mm Hg > 5 minutes, n (%)	2 (18)	0	0.002
Heart rate < 30 beats.min-1> 1 minute, n (%)	0	0	
Systolic blood pressure<60 mmHg > 5 minutes, n (%)	1 (9)	2 (4)	

Abbreviations: IQR = interquartile range; SD = standard deviation; Vt = tidal ventilation; PBW = predicted body weight; Pplat = plateau airway pressure; Pdrive = driving pressure; PEEP = positive end expiratory pressure; Early prone was defined as prone positioning started within 36 hours after intubation; Pressure ulcers which indicated the requirement of surgical treatment was collected.

The parameters of lung-protective ventilation did not differ between the two groups. The achievement of early prone positioning increased from 56% to 98% in the post-introduction group, and the average duration of prone positioning significantly increased from 15 h to 18 h in the post-introduction group (*P* = 0.007). Although the duration of prone positioning increased, there was no increase in the number of complications in the post-introduction group.

### Primary and secondary outcomes

The results of outcomes are shown in Table 3.

**Table 3:** Primary and secondary outcomes.

	Overall (n = 68)	Pre-introduction (n = 18)	Post-introduction (n = 50)	Odds Ratio with post-introduction [95%Cl]	P Value
Mortality, no./total no. (%)					
at ICU	5/68 (7)	3/18 (17)	2/50 (4)	0.214 [0.016–2.053]	0.111
The duration of invasive mechanical ventilation, median {IQR}	11 {7–17}	15 {8–35}	10 {6–13}		0.039
adjusted					0.060
The length of ICU stay, median {IQR}	13 {8–31}	26 {16–37}	11 {7–16}		<0.001
adjusted					0.003

Abbreviations: IQR = interquartile range; VAP = ventilator-associated pneumonia; ICU = intensive care unit

The mortality rate in the ICU showed a decreasing trend from 17% to 4%; however, it did not show statistical significance (OR, 0.21; 95% Cl, 0.01–2.05).

The duration of ventilation was reduced from 15 days to 10 days, with statistical significance in the univariate analysis; however, it did not remain statistically significant after adjusting for age, sex, and SOFA score (S4 Table).

The length of stay in the ICU was reduced from 33 to 11 days, with statistical significance. Even after adjusting for age, sex, and SOFA score, the differences remained statistically significant (*P* = 0.003, S5 Table).

## Discussion

### Overview

The introduction of the protocol for COVID-19 pneumonia requiring invasive ventilation did not improve mortality in the ICU; however, it reduced the length of ICU stay and tended to shorten the duration of ventilation.

### Mortality

In our study, mortality at the ICU did not improve by introducing the protocol.

This may be because the rate of V-V ECMO tended to be higher in the pre-introduction group. The results of V-V ECMO treatments for COVID-19 pneumonia have been good, according to a report by the Extracorporeal Life Support Organization [22]. The high rate of V-V ECMO could have positively affected mortality in the pre-introduction group. However, it is important to note that despite the lack of difference in severity between the two groups, the rate of V-V ECMO was reduced by introducing the protocol, and ICU mortality was not worse in the post-introduction group.

In addition, ICU mortality tended to be lower in our hospital than reported in previous studies. For example, the overall mortality of 7% in the ICU in our cohort was lower compared with 38% reported in the PRoVENT-COVID cohort (Table 3) [23]. The small number of death events may have contributed to the lack of statistically significant differences.

Therefore, it is important that the introduction of the protocol did not worsen mortality with no increase in V-V ECMO initiation.

### Length of ICU stay and duration of ventilation

The protocol reduced the length of ICU stay and ventilation duration.

In our study, despite no difference in the rate of achievement of lung-protective ventilation in the two groups, the achievement of early applied prone positioning and the duration of prone positioning increased significantly, suggesting that these approaches could be effective. In the PROSEVA study, the achievement of early prone positioning and the duration of prone positioning reduced the length of stay in the ICU and duration of ventilation [7]. Therefore, the results of our study are consistent with those of the PROSEVA study. As a result, the introduction of the protocol could comply with these approaches, contributing to an improvement in the length of stay in the ICU and the duration of ventilation.

### Stratification of patients in whom the protocol was effective

Mortality rates may differ depending on the responsiveness of the PaO_2_/ F_I_O_2_ ratio after prone positioning [24]. In a previous study, patients whose PaO_2_/ F_I_O_2_ ratio increased by 20 mmHg or more after prone positioning had a lower mortality rate than those whose ratio changed by less than 20 mmHg (38% vs. 65%, p = 0.039) [24].

However, our study did not examine the assessment of respiratory status before and after prone positioning, and stratification may alter the validity of the protocol.

### Adverse events associated with the protocol

There were no significant differences between the groups with respect to adverse events associated with prone positioning in our study, as well as in the PROSEVA study [7]. The low incidence of adverse events could be related to the fact that our hospital and the facilities that participated in PROSEVA study were familiar with the management of severe respiratory failure. No other protocol-related adverse events were found.

### Limitation

In addition to our study design being single-centred and retrospective, the severe acute respiratory syndrome coronavirus 2 (SARS-CoV-2) mutation, prevalence and susceptible age of COVID-19, and drug treatment strategy for COVID-19 pneumonia changed significantly before and after the introduction of the protocol. These could have influenced the results, and the number of cases was not sufficient to adequately adjust for the confounding factors.

Despite these limitations, during a pandemic, this protocol could contribute to securing medical resources by reducing the length of ICU stay and duration of ventilation.

With no increasing adverse events, this protocol could be generalised since it does not require special drugs or equipment but only prone positioning.

## Conclusions

The introduction of the protocol for patients with COVID-19 pneumonia who required invasive ventilation was associated with improved length of stay in the ICU and duration of ventilation; however, the difference was not statistically significant. Nevertheless, the protocol could contribute to the efficient use of medical resources during the pandemic through adherence to lung protection strategies. Further investigation regarding the efficacy of the protocol using a multicentre prospective study is required.

## Supporting information

S1 Table STROBE statement—Checklist of items that should be included in reports of cohort studies. (DOCX)

S2 Table Contraindication for prone positioning. a. Intracranial pressure >30 mmHg or cerebral perfusion pressure <60 mmHg. b. Massive haemoptysis requiring an immediate surgical or interventional radiology procedure. c. Tracheal surgery or sternotomy during the previous 15 days. d. Serious facial trauma or facial surgery during the previous 15 days. e. Deep venous thrombosis treated for less than 2 days. f. Cardiac pacemaker inserted in the last 2 days. g. Unstable spine, femur, or pelvic fractures. h. Mean arterial pressure lower than 65 mmHg. i. Pregnant women. j. Single anterior chest tube with air leaks.
(PPTX)

S3 Table Drug administration strategy in the study protocol. Medications for coronavirus disease 2019 (COVID-19) pneumonia were as follows: (i) Immune-based agent: We administered dexamethasone, dose was 0.15 mg/kg, and the duration of administration was from 7 days to 10 days [25]. (ii) Antiviral agent: Remdesivir was administered at a dose of 200 mg on the first day and 100 mg thereafter; the duration of administration was from 7 days to 10 days [26]. (iii) Thromboprophylaxis agent: We administered subcutaneous low-molecular-weight heparin 10,000 units until the patients could move automatically [27]. In addition, as a preventative antibiotic for ventilator-associated pneumonia (VAP), we chose tazobactam-piperacillin based on previous reports regarding the frequency of VAP occurrence due to the causative bacteria and used it until the patient was extubated [20].
(PPTX)

S4 Table Multiple linear regression analysis of duration of ventilation. The multivariable analysis was conducted with age, sex, and sequential organ failure assessment (SOFA): these are considered risk factors for severe coronavirus disease 2019 (COVID-19) pneumonia. The duration of ventilation was log-transformed before the multivariate analysis. The pre-introduction group included 17, and the post-introduction group included 50 patients.
(PPTX)

S5 Table Multiple linear regression analysis of length of intensive care unit (ICU) stay. The multivariable analysis was conducted with age, sex, and sequential organ failure assessment (SOFA): these are considered risk factors for severe severe coronavirus disease 2019 (COVID-19) pneumonia. The length of ICU stay was log-transformed before the multivariate analysis. The pre-introduction group included 18 patients, and the post-introduction group included 50 patients.
(PPTX)
