# Supplementary figures and images for: Effect of a systematic lung-protective protocol for COVID-19 pneumonia requiring invasive ventilation: A single center retrospective study
Source: PLoS One. 2023 Jan 12;18(1):e0267339. doi: 10.1371/journal.pone.0267339 (PMC9836282; doi:10.1371/journal.pone.0267339)

## Slide 1
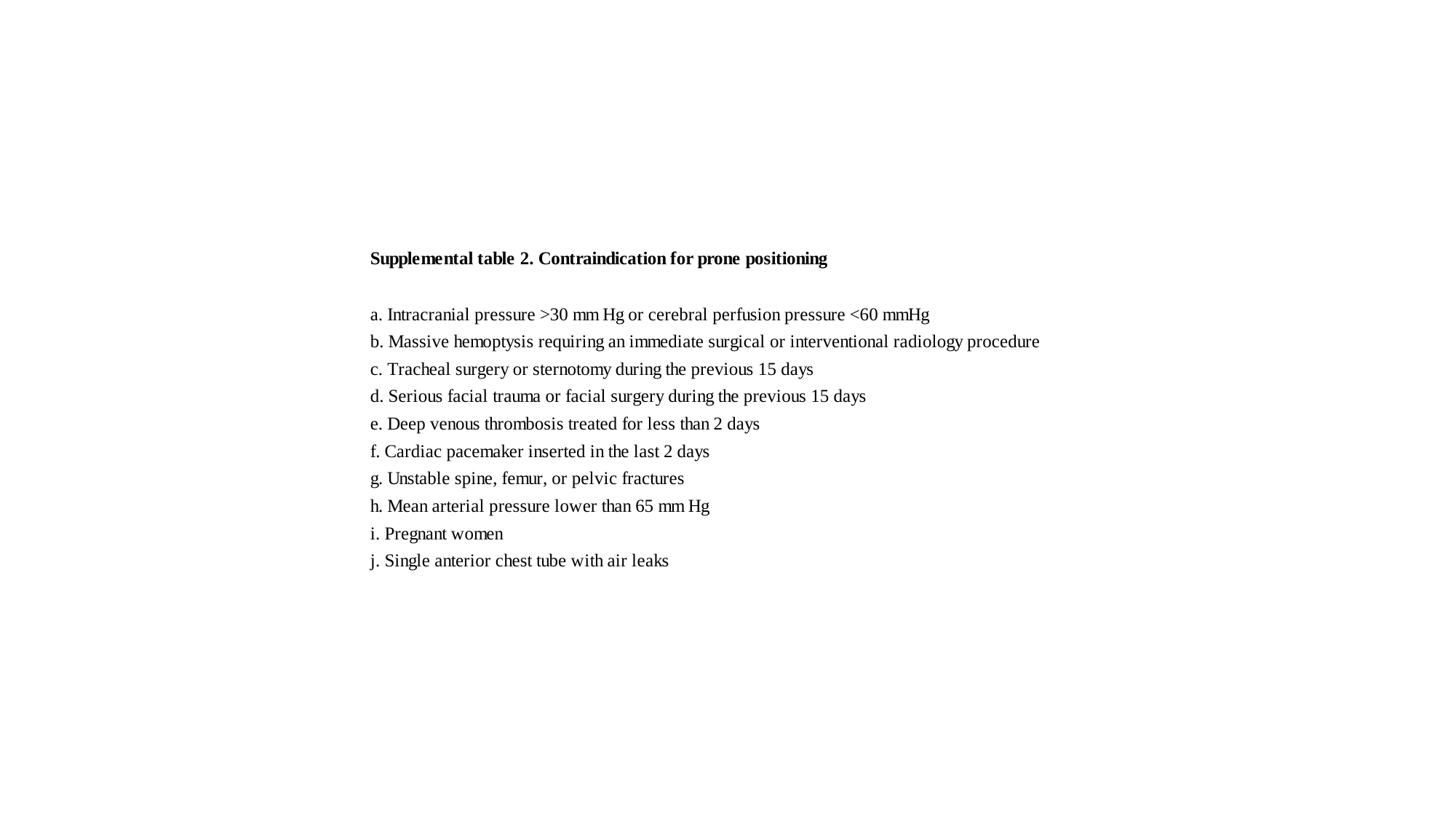

Supplement: S2 Table — a. Intracranial pressure >30 mmHg or cerebral perfusion pressure <60 mmHg. b. Massive haemoptysis requiring an immediate surgical or interventional radiology procedure. c. Tracheal surgery or sternotomy during the previous 15 days. d. Serious facial trauma or facial surgery during the previous 15 days. e. Deep venous thrombosis treated for less than 2 days. f. Cardiac pacemaker inserted in the last 2 days. g. Unstable spine, femur, or pelvic fractures. h. Mean arterial pressure lower than 65 mmHg. i. Pregnant women. j. Single anterior chest tube with air leaks. (PPTX) [file pone.0267339.s002.pptx]

## Slide 1
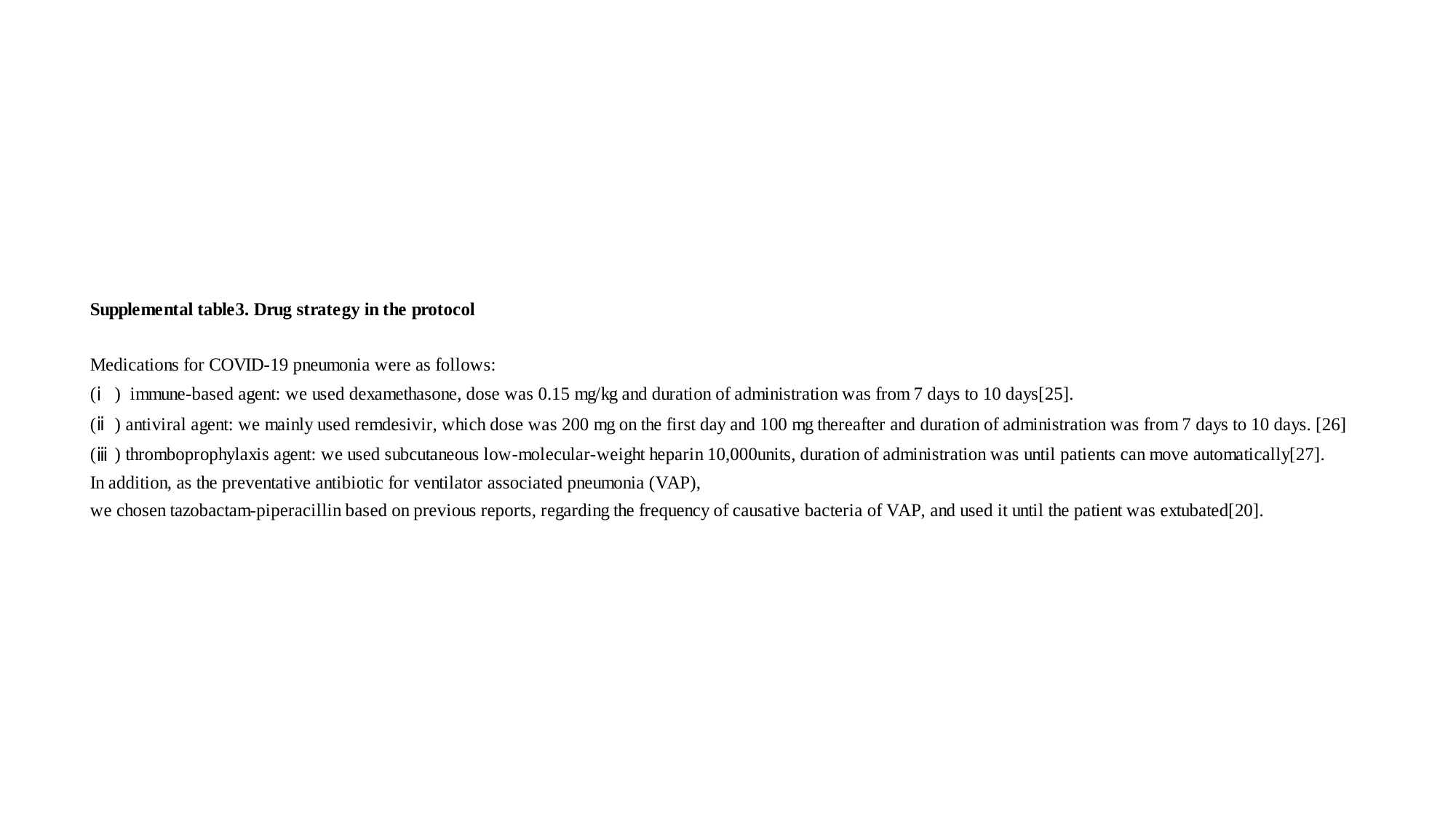

Supplement: S3 Table — Medications for coronavirus disease 2019 (COVID-19) pneumonia were as follows: (i) Immune-based agent: We administered dexamethasone, dose was 0.15 mg/kg, and the duration of administration was from 7 days to 10 days [25]. (ii) Antiviral agent: Remdesivir was administered at a dose of 200 mg on the first day and 100 mg thereafter; the duration of administration was from 7 days to 10 days [26]. (iii) Thromboprophylaxis agent: We administered subcutaneous low-molecular-weight heparin 10,000 units until the patients could move automatically [27]. In addition, as a preventative antibiotic for ventilator-associated pneumonia (VAP), we chose tazobactam-piperacillin based on previous reports regarding the frequency of VAP occurrence due to the causative bacteria and used it until the patient was extubated [20]. (PPTX) [file pone.0267339.s003.pptx]

## Slide 1
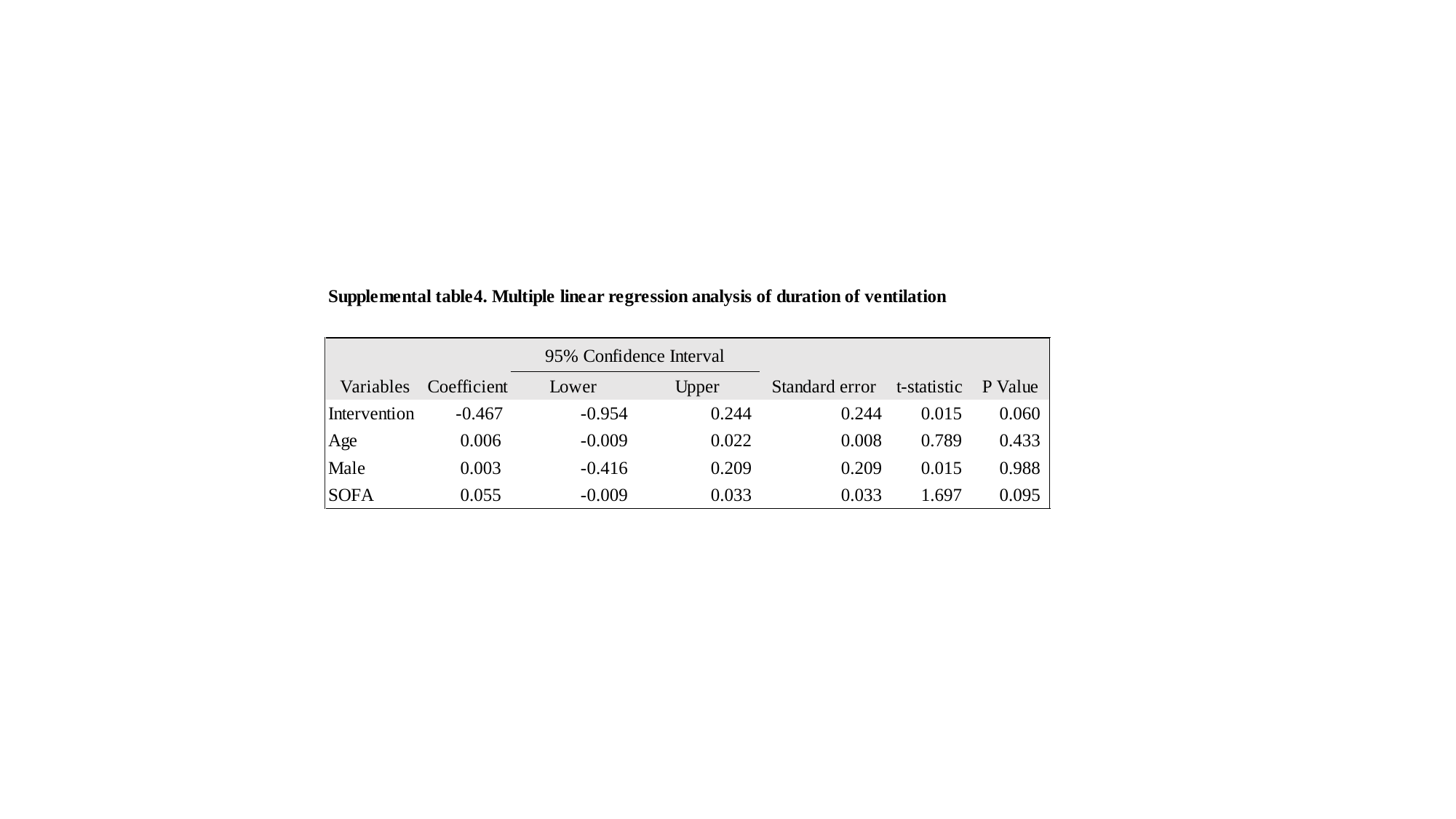

Supplement: S4 Table — The multivariable analysis was conducted with age, sex, and sequential organ failure assessment (SOFA): these are considered risk factors for severe coronavirus disease 2019 (COVID-19) pneumonia. The duration of ventilation was log-transformed before the multivariate analysis. The pre-introduction group included 17, and the post-introduction group included 50 patients. (PPTX) [file pone.0267339.s004.pptx]

## Slide 1
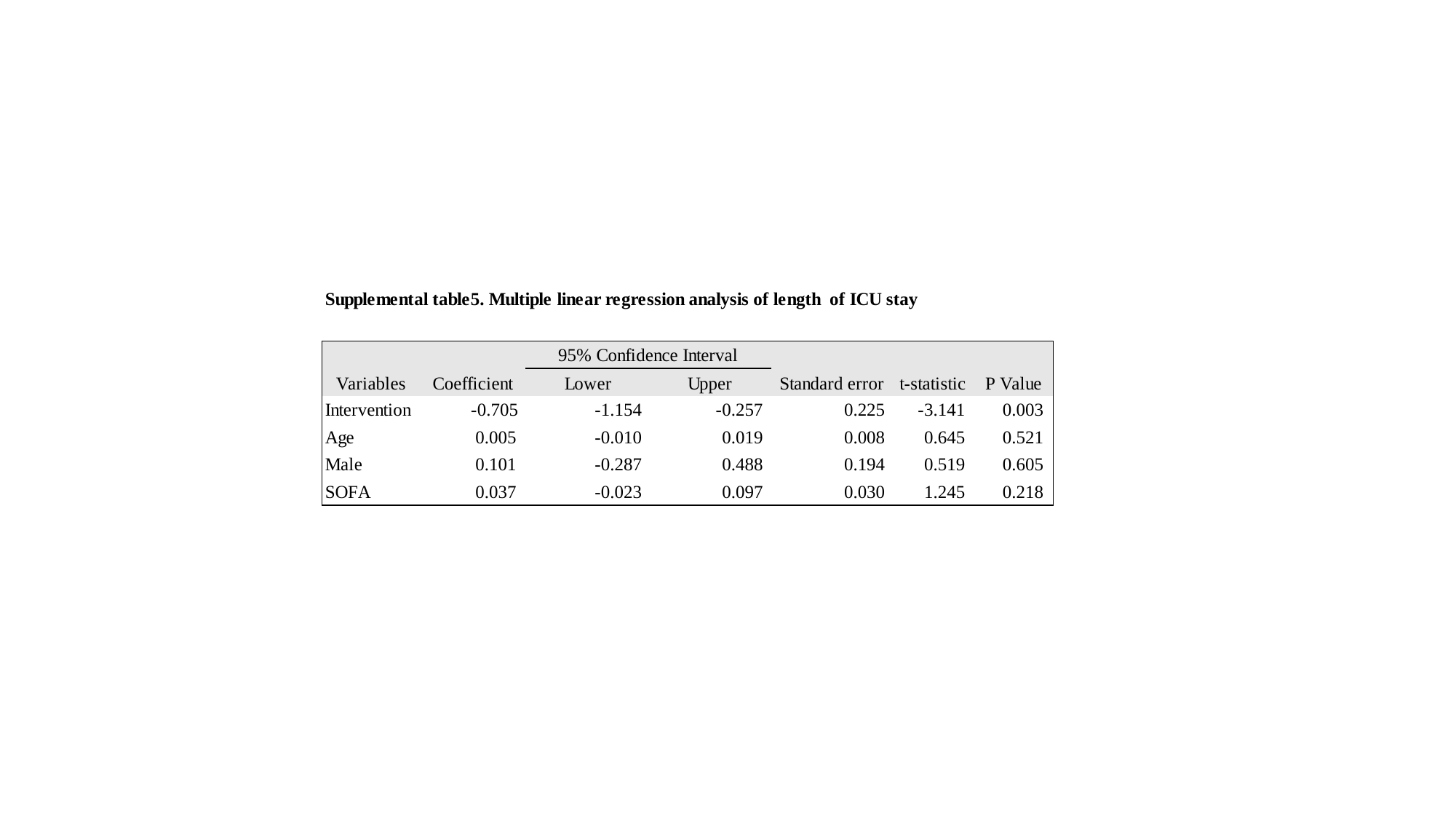

Supplement: S5 Table — The multivariable analysis was conducted with age, sex, and sequential organ failure assessment (SOFA): these are considered risk factors for severe severe coronavirus disease 2019 (COVID-19) pneumonia. The length of ICU stay was log-transformed before the multivariate analysis. The pre-introduction group included 18 patients, and the post-introduction group included 50 patients. (PPTX) [file pone.0267339.s005.pptx]
